# Cell and extracellular vesicle therapies for AKI in critical care: clinical translation, organ-support integration, and lessons learned

**DOI:** 10.3389/fmed.2026.1818060

**Published:** 2026-07-09

**Authors:** Amankeldi A. Salybekov, Aiman Kinzhebay, Aina Zhanymbetova, Shuzo Kobayashi

**Affiliations:** 1 Kidney Disease and Transplant Center, Shonan Kamakura General Hospital, Kamakura, Japan; 2 Regenerative Medicine Division, Cell and Gene Therapy Department, Qazaq Institute of Innovative Medicine, Astana, Kazakhstan; 3 Department of Oncology, Astana Medical University, Astana, Kazakhstan

**Keywords:** acute kidney injury, critical care nephrology, extracellular vesicles, regenerative medicine, stem cells

## Abstract

Acute kidney injury (AKI) affects up to half of critically ill intensive care unit (ICU) patients and remains associated with high mortality despite advances in renal replacement therapy (RRT). Current management is largely supportive and does not modify the underlying biological mechanisms driving inflammation, microvascular dysfunction, and maladaptive repair. Regenerative approaches using mesenchymal stromal cells (MSCs) and extracellular vesicles (EVs) have emerged as potential adjunctive therapies aimed at promoting renal recovery beyond organ support. Preclinical studies consistently demonstrate that MSCs and MSC-derived EVs reduce inflammation, attenuate oxidative stress, preserve tubular integrity, and enhance microvascular repair through paracrine signaling. However, early clinical trials in post-operative, sepsis-associated AKI populations have shown safety but limited efficacy signals. These neutral outcomes likely reflect AKI heterogeneity, delayed intervention timing, non-enriched patient selection, and reliance on insensitive endpoints such as serum creatinine. EV-based therapies offer practical advantages over whole-cell approaches, including scalable manufacturing, off-the-shelf availability, and easier integration into ICU workflows. Future progress requires phenotype-enriched enrollment strategies, biomarker-guided therapeutic windows, harmonized EV dosing and potency assays, and kidney-specific endpoints such as dialysis-free days.

## 1 Why ICU-AKI needs regenerative options

Acute kidney injury (AKI) affects up to 50% of critically ill intensive care unit (ICU) patients, significantly increasing morbidity and mortality despite advances in renal replacement therapy (1). Current standard care, limited to renal replacement therapy (RRT) and supportive measures, fails to modify the underlying disease process or improve survival rates, creating a critical unmet need for therapies promoting renal repair and regeneration (2, 3). This mini-review examines cell-based therapies and extracellular vesicles (EVs) as adjunctive regenerative strategies that modulate inflammation and enhance tubular recovery beyond conventional organ support (1, 4).

While existing literature extensively reviews the preclinical efficacy of cellular secretomes in isolated renal injury models, the unique innovation of this mini-review lies in its deliberate focus on the operational and biophysical intersection of regenerative therapeutics with active intensive care workflows. Specifically, we synthesize how the complex molecular payloads of mesenchymal stromal cells (MSCs) and EVs interact with extracorporeal organ-support platforms, such as continuous renal replacement therapy (CRRT) and extracorporeal membrane oxygenation (ECMO), offering a pragmatic roadmap for real-world clinical integration rather than a mere restatement of benchside biology.

### 1.1 Burden and unmet need in critical care nephrology

Etiology of AKI in the ICU ranges from sepsis-associated syndromes to ischemia-reperfusion injury, resulting in heterogeneity that conventional interventions fail to address at a mechanistic level (5). Such variability complicates determining the optimal timing for RRT initiation. Recent randomized controlled trials have failed to show consistent survival benefits for early initiation and suggest that starting therapy without clear necessity may be harmful (6, 7). Current biomarkers similarly lack the ability to distinguish patients likely to recover spontaneously from those requiring dialysis (8). Despite the high prevalence of AKI among critically ill patients, no approved pharmacological therapies exist to prevent injury or actively enhance renal recovery (3, 6). This lack of progress partially stems from the difficulty of precisely defining the timing, etiology, and biological phase of AKI in the ICU, where clinically similar presentations may reflect fundamentally different underlying mechanisms (6, 9). Consequently, current classification systems, based largely on functional criteria, fail to capture the biological heterogeneity that drives divergent trajectories and outcomes (9, 10).

This limitation has important therapeutic implications, as broad, “one-size-fits-all” approaches obscure distinct injury and repair endotypes, contributing to inconsistent responses to interventions and repeated failures of disease-modifying strategies in critical illness (5–7). AKI encompasses at least three biologically distinct endotypes with differing regenerative potential: ischemia-reperfusion injury, characterized by tubular epithelial cell death and microvascular dysfunction amenable to pro-repair signaling; sepsis-associated AKI, driven predominantly by inflammatory microcirculatory injury and mitochondrial dysfunction rather than overt tubular necrosis; and nephrotoxic AKI, where direct tubular toxicity and oxidative stress predominate (5–7). These distinctions matter therapeutically, as MSC-derived EVs have demonstrated differential efficacy across these contexts in preclinical models, with stronger pro-regenerative responses observed in ischemic models compared to septic AKI, where the inflammatory environment may alter EV cargo uptake and downstream signaling (2). Crucially, this heterogeneity extends beyond clinical etiology into molecular subphenotypes (e.g., hyper-inflammatory vs. hypo-inflammatory endotypes) identified through transcriptomic and protein biomarker profiling (6). Dissecting these distinct biological trajectories is vital for clinical translation, as a hyper-inflammatory subphenotype may require heavily immunomodulatory EV cargo, whereas an adaptive, metabolic-stalled phenotype would benefit more from bioenergetic support (6). Regenerative approaches modulate injury cascades and promote repair processes beyond RRT excretory support (1, 3, 5). Their potential benefit is greatest when interventions are deployed early and aligned with the dominant underlying pathophysiology, facilitating a shift from passive support to active renal regeneration (1, 5).

### 1.2 Rationale and challenges of regenerative approaches as adjuncts to organ support and standard care

The shift to regenerative medicine acknowledges that supportive measures like CRRT, fluid management, vasopressors, and nephrotoxin avoidance manage metabolic consequences without addressing cellular damage, dysregulated immunity, or stalled repair, driving CKD (chronic kidney disease) progression (3, 5, 8). Trials like AKIKI (Artificial Kidney Initiation in Kidney Injuries) and STARRT-AKI (Standard versus Accelerated Initiation of Renal Replacement Therapy in Acute Kidney Injury) confirm CRRT’s lack of survival or recovery benefits, limiting it to supportive care (8, 9). In contrast, cell therapies and EVs via paracrine mechanisms modulate inflammation, promote angiogenesis, and inhibit fibrosis as adjuncts to CRRT and standard care, addressing multifactorial repair (1, 10). Preclinical evidence shows MSCs and EVs release growth factors, chemokines, and cytokines that induce proximal tubular proliferation, dedifferentiation, and angiogenesis, restoring parenchyma and reversing hemodynamic and structural damage (11).

However, completing this therapeutic rationale requires overcoming barriers unique to the critical care environment. Clinical translation of MSC- and EV-based therapies in the ICU is limited by variable biodistribution, reduced target-organ delivery, and uncertainty in dosing and timing under conditions of critical illness (1, 12). AKI heterogeneity further demands precision medicine tailoring to pathophysiology and patient responses (6). Extracorporeal therapies like ECMO alter pharmacokinetics through changes in distribution, clearance, and adsorption, necessitating CRRT-cycle dose adjustments (5, 13–15). In addition, trials of recombinant alkaline phosphatase in sepsis-AKI highlight the difficulty of modulating established inflammation, emphasizing the need for early intervention before irreversible failure (5). Accordingly, future research requires biomarkers to define therapeutic windows (5, 16). Given ICU patient diversity and multi-organ failure obscuring benefits (17), trials require: (1) cohort enrichment and sub-phenotyping (7); (2) refined CRRT integration (18); (3) multi-omics or AI phenotyping (8, 16); (4) composite endpoints like MAKE-90 (Major adverse kidney events at 90 days) (17); (5) mechanism-endotype matching via biomarkers (6, 7, 19, 20); (6) rapid point-of-care multi-omic assays (6); (7) renal reserve and dynamic monitoring of regenerative capacity in persistent AKI (5). Preclinical and translational studies further inform these strategies. Advanced models validate EV dosing and timing, preventing AKI-to-CKD via maladaptive repair inhibition (10, 21, 22). Single-cell transcriptomics identifies fibrosis-driving “failed-repair” populations, targeting IGF-1/Bcl-2 pathways (10, 22).

Despite these advances, major translational gaps include standardized assessment of AKI-to-CKD progression and scalable EV manufacturing (22, 23). Clinical application will require standardized production protocols, potency assays, and quality control (22). Production can be enhanced via hypoxia, bioreactors, hollow fibers (24), but ultracentrifugation limits scalability due to contaminants/aggregation (1, 25). These developments position MSCs and their EVs as primary regenerative modalities for ICU renal recovery, as “off-the-shelf” therapies for acute intervention (26, 27). There is growing recognition that paracrine signaling, rather than the engraftment and differentiation of administered cells into renal parenchyma, drives their therapeutic effects (1). Consequently, therapeutic focus has shifted toward the secretome, particularly EVs, which avoid risks of whole-cell administration while retaining the capacity to regulate critical pathways (28, 29). By transferring microRNAs and proteins to target cells, these vesicles modulate cell fate and enhance renal tubular cell survival to actively promote tissue regeneration rather than mere exogenous organ support (30, 31).

Recent work has further extended EV engineering beyond native cargo, for example, Grignano et al. demonstrated that MSC-derived EVs loaded with ATP via microfluidic technology restored intracellular energy levels in ischemic renal tubular cells *in vitro*, addressing the rapid ATP depletion that characterizes early ischemic AKI and that conventional exogenous ATP delivery cannot overcome due to hydrolysis and poor membrane permeability (32).

## 2 What to carry forward from preclinical work

### 2.1 The consistent effects across experimental AKI models

**FIGURE 1:**
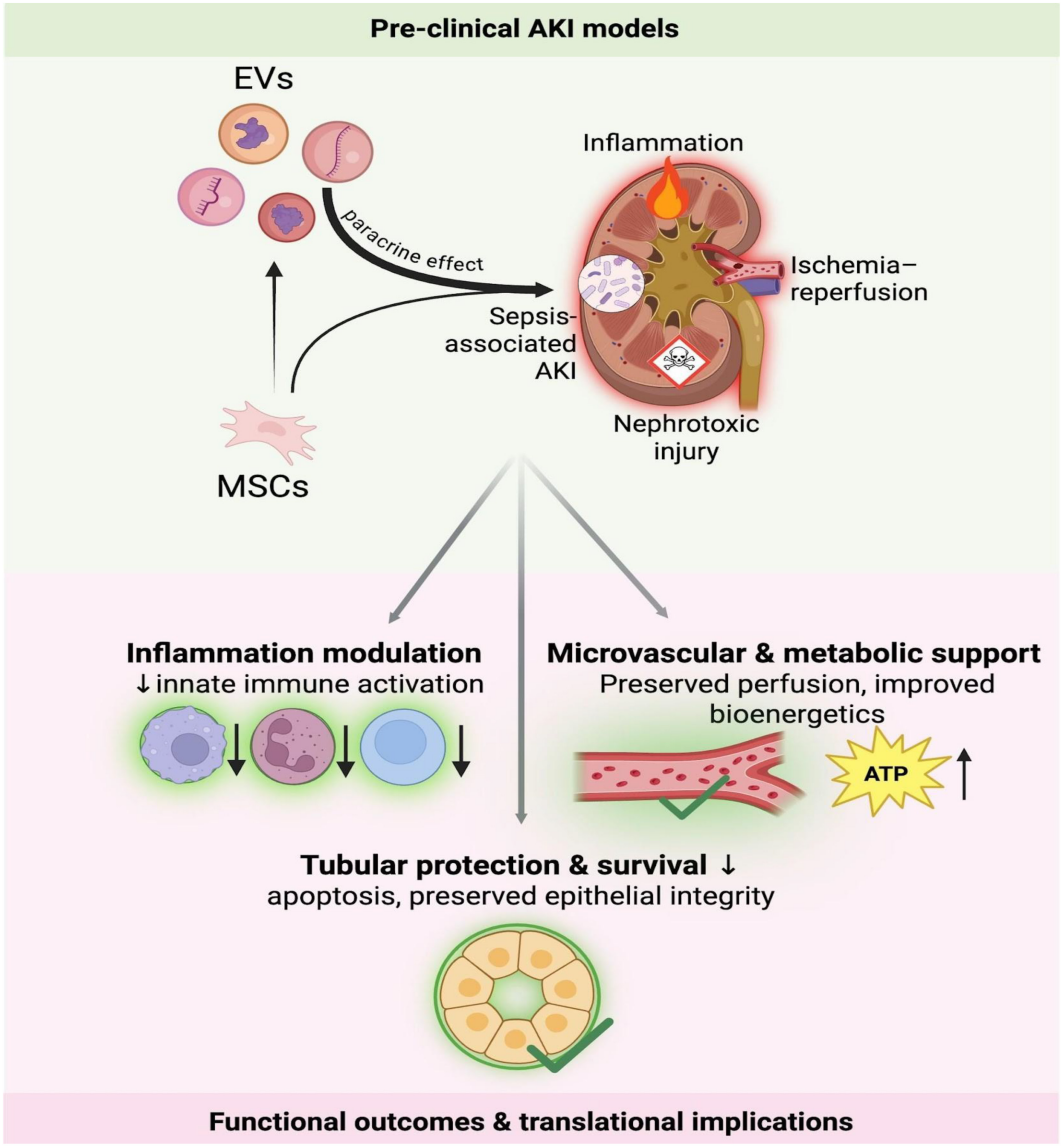
Pre-clinical studies across ischemic, septic, and toxic models of acute kidney injury consistently demonstrate that MSCs and their EVs modulate inflammation, protect tubular integrity, and support microvascular and metabolic function.

Preclinical studies show MSCs and secreted EVs promote functional recovery, modulate inflammation, and provide tubular protection via bioactive factors like IGF-1, FGF-2, and TGF-β (1, 33). These regenerative effects are observed in ischemia-reperfusion and nephrotoxic models, where these therapies reduced serum creatinine levels, attenuated histological damage, and improved survival rates (1, 34). Meta-analyses of animal studies support that stem/progenitor cell-derived EVs significantly reduce serum creatinine and markers of tubular and endothelial injury while enhancing tubular proliferation (2). Furthermore, these vesicles have been shown to improve renal recovery, limit progression of injury, and reduce fibrosis in animal models of AKI (35). The therapeutic efficacy of EVs is enhanced by preconditioning strategies like hypoxia, amplifying tissue repair capabilities in renal ischemic injury models (27). The main preclinical mechanisms of MSC/EV-mediated renal protection are summarized in Figure 1.

Mechanistically, MSC-EVs exert cytoprotective, regenerative, and immunomodulatory effects through the transfer of proteins, growth factors, and microRNAs that promote cell survival, proliferation, and angiogenesis (1, 11). EV cargo activates antioxidant and anti-apoptotic pathways, including Nrf2/ARE (nuclear factor erythroid 2-related factor 2 / antioxidant response element) signaling, preserves mitochondrial function, and maintains redox homeostasis (1, 36, 37). For example, EV-mediated transfer of IGF-1 receptors enhances Nrf2 activation and accelerates tubular repair, while increased hepatocyte growth factor expression activates Erk1/2 signaling to promote tubular dedifferentiation and growth (1, 11). In parallel, MSC-EVs suppress pro-inflammatory pathways such as NF-κB signaling and modulate immune cell infiltration and cytokine production, creating a microenvironment favorable for regeneration (1, 38, 39). The therapeutic efficacy of EVs can be further enhanced by preconditioning strategies such as hypoxia or melatonin, which amplify reparative and antioxidant responses (36). Together, these reproducible functional and mechanistic effects across diverse injury models indicate convergence on common pathways of inflammation control, oxidative stress reduction, and tubular repair, supporting the consistent renoprotective signals observed with MSC-EV therapy.

### 2.2 From engraftment to paracrine and secretome-mediated mechanisms

The conceptual framework of regenerative therapies has shifted from the assumption that administered MSCs engraft and differentiate into renal tubular cells to replace damaged tissue toward a predominantly paracrine mechanism. This shift is supported by observations that the transient presence of administered cells in the kidney cannot account for the rapid and sustained improvement in renal function (39). This has led researchers to identify the secretome, particularly EVs carrying microRNAs, proteins, and lipids, as the primary mediator of tissue repair and immunomodulation (39). EVs facilitate intercellular communication by shuttling functional cargoes (proteins, lipids, nucleic acids) to target cells, reducing tissue injury and promoting repair and regeneration (2). For instance, bone marrow-derived mesenchymal stromal cell EVs have been shown to alleviate cisplatin-induced cell injury by inhibiting hsp70-mediated NLRP3 inflammasomes (1). Meanwhile, in glycerol-induced myolysis models, these vesicles deliver specific mRNAs that influence cell cycle progression and promote proliferation through growth factors (1). It is important to acknowledge that EVs derived from MSCs of different tissue origins including bone marrow, adipose tissue, umbilical cord, and Wharton’s jelly exhibit significant heterogeneity in surface marker profiles, cargo composition, and immunomodulatory potency. Source-dependent differences in miRNA repertoire, growth factor content, and biogenesis pathways may lead to divergent therapeutic outcomes across experimental and clinical settings (26). Unless otherwise specified, findings summarized in this review derive from studies using MSCs of heterogeneous origins, and this variability should be considered when interpreting comparative preclinical and clinical data.

### 2.3 Translational parameters: timing window, AKI phenotype context, delivery route constraints in ICU

Successful translation of regenerative therapies from bench to bedside requires careful consideration of parameters unique to the intensive care environment: optimal timing relative to injury onset, AKI phenotype, and delivery routes in critically ill patients. Systemic intravenous administration is most feasible for CRRT patients, but preclinical studies indicate local delivery may provide superior anti-fibrotic protection (40–42). ICU constraints favor intravenous routes despite pulmonary first-pass trapping of cells and rapid EV clearance (40–42). Intra-arterial delivery enhances renal uptake in cisplatin models, but risks like bleeding limit its use (1, 42). Emerging strategies include engineering vesicles with targeting peptides (e.g., Arg-Gly-Asp motifs for integrin αVβ3 binding) to improve homing (12).

Defining a precise therapeutic window is crucial, as early administration enables internalization by healthy cells, upregulating stress-resistance genes, while delayed dosing limits efficacy due to ATP depletion (12, 22). AKI heterogeneity demands phenotype-specific optimization, as sepsis differs from ischemic or nephrotoxic insults in biodistribution and response (31, 40).

## 3 Clinical evidence: where we truly are

Cell-based therapies have been explored in the context of AKI for more than a decade, largely driven by encouraging results from experimental models. Preclinical studies consistently showed that cell-based or cell-derived approaches modulate immune responses, attenuate systemic inflammation, and preserve the renal microvasculature. Moving from experimental settings to real-world clinical practice has proved far more difficult than initially anticipated. Across a wide range of AKI populations, clinical studies have struggled to demonstrate a consistent and reproducible therapeutic effect. This has remained true even in trials where regenerative approaches were implemented as planned and were not associated with major safety concerns (43, 44). Table 1 summarizes selected clinical trials of whole-cell regenerative therapies for AKI.

**TABLE 1:** Selected clinical trials of cell-based and stem cell regenerative therapies for acute kidney injury.

Trial ID	Study title	Phase / type	Population	Intervention	Primary endpoint	Status / key findings
jRCTb0 30190231 Japan (jRCT)	Autologous G-CSF-mobilized peripheral blood CD34 + cell transplantation for severe AKI	Phase I/II Interventional Dose-escalation	Severe AKI requiring hemodialysis; ischemic/ hypertensive etiology	Autologous CD34 + cells infused directly into renal arteries; three dose levels (5 × 10^5^, 1 × 10^6^, 2 × 10^6^/kg body weight) [a]	Primary: Safety Secondary: Efficacy (serum creatinine, eGFR)	[Active] First-in-human report (46): no major adverse events; serum creatinine improved from 7.56 to 2.96 mg/dL at 23 weeks; transient fever and thrombocytosis noted
NCT066 54193 Clinical Trials.gov	Allogeneic HB-adMSCs vs. Placebo for the Treatment of Acute Kidney Injury	Not specified Interventional RCT	Trauma-induced AKI	Allogeneic adipose-derived MSCs (HB-adMSCs) vs. normal saline placebo; IV administration	Safety and effectiveness; prevention of AKI progression	[Recruiting] No results available; ongoing enrollment across three US academic centers (UT Houston, UCSF, UAB)
NCT071 01913 Clinical Trials.gov	Occurrence of AKI after CAR-T Cell Treatments in B-cell Lymphoma	N/A Observational [b]	B-cell lymphoma patients receiving CAR-T cell therapy; subset with baseline CKD	CAR-T cell therapy (CD19-targeted); AKI incidence and risk factors observed prospectively	Incidence of AKI post-CAR-T; risk factor identification; long-term kidney function trajectory	[Active] No results available; provides insight into cell therapy-associated nephrotoxicity in the ICU context
NCT007 33876 Clinical Trials.gov	Prevention and treatment of post-operative AKI with allogeneic mesenchymal stem cells in patients requiring on-pump cardiac surgery	Phase I Interventional Dose-escalation	High-risk on-pump cardiac surgery patients (age > 65; underlying CKD, DM, CHF, COPD, or hypertension)	Allogeneic bone marrow–derived MSCs infused via femoral catheter into suprarenal aorta; three escalating dose levels (*n* = 5 per cohort; 16 total enrolled)	Primary: Safety Secondary: AKI incidence, frequency/severity/ duration, dialysis dependency, length of stay, 30-day mortality vs. historical controls	[Completed] Gooch et al. (47): no serious adverse events attributable to MSCs; AKI incidence 0% in MSC group vs. ∼20% in matched historical controls; reduced hospital length of stay and readmission. Results uncontrolled (no randomized comparator group). Served as basis for Phase II ACT-AKI trial.
NCT016 02328 Clinical Trials.gov (ACT-AKI)	Allogeneic mesenchymal stem cells (AC607) vs. placebo for treatment of AKI after cardiac surgery	Phase II Interventional Multicenter RCT	Adults with early post-operative AKI following cardiac surgery; screened from 26,548 patients across 27 US centers	Allogeneic BM-derived MSCs (AC607) injected into aorta above renal artery vs. placebo (*n* = 156 randomized, 2012–2014)	Time to post-intervention creatinine return to baseline; secondary: dialysis requirement, 30-day mortality, adverse events	[Completed] Swaminathan et al., JASN 2018: primary outcome showed no significant difference vs. placebo. Secondary outcomes trended numerically toward placebo. Non-significant higher incidence of dialysis or mortality in MSC arm. Concluded MSC therapy may be more effective for AKI prevention than treatment; clinical complexity post-cardiac surgery may limit MSC efficacy.
NCT012 75612 Clinical Trials.gov (CIS/MS C08)	*Ex-vivo* expanded mesenchymal stem cells to repair the kidney in cisplatin-induced acute renal failure in patients with solid organ cancers	Phase I Interventional single-group [c]	Solid organ cancer patients developing AKI after cisplatin chemotherapy	Single IV infusion of allogeneic donor ex-vivo expanded MSCs (1 × 10^6^/kg); dose escalation in subsequent cohort if feasible and safe	Feasibility and safety of systemic MSC infusion; secondary: renal function improvement	[Withdrawn] Sponsor (Mario Negri Institute / Remuzzi group): withdrawn due to inability to enroll, patients evaluated did not meet the protocol-specified AKI severity criterion. No efficacy data generated. Notable as the first attempt to translate MSC therapy to chemotoxic (non-ischemic) AKI in a clinical setting.
NCT030 15623 Clinical Trials.gov (SBI-101)	A study of cell therapy for subjects with acute kidney injury who are receiving continuous renal replacement therapy	Phase I/II Interventional Dose-escalation	AKI patients receiving continuous renal replacement therapy (CRRT); underlying systemic inflammation	SBI-101: BM-MSCs immobilized in extracapillary space of hollow-fiber hemofiltration device; circulating blood exposed to MSC paracrine factors *ex vivo* [d] Low: 250 × 10^6^ cells; High: 750 × 10^6^ cells	Primary: safety/tolerability (IP-related SAEs) Secondary: anti-inflammatory biomarkers (TNF-α, IFN- γ, IL-10, TGF-β1)	[Completed] Swaminathan et al. (48) (Stem Cells Transl. Med.): *n* = 16 (12 treatment, 4 sham); no serious adverse events. Anti-inflammatory effects confirmed: ↓TNF-α, ↓IFN-γ; ↑IL-10, ↑TGF-β1 vs. sham. Circuit clotting noted as limitation requiring anticoagulation. Safety-focused; clinical efficacy not assessed at this stage.
NCT041 94671 Clinical Trials.gov	Clinical Trial of mesenchymal stem cells in the treatment of severe acute kidney injury	Not specified Interventional	Severe AKI patients	MSC infusion; dose: 10^6^ cells/kg body weight; IV administration	Primary: eGFR	[Unknown] Beijing, China. No published results identified. Listed as “Unknown” status in registry. Cited in Salybekov et al. (4) as an ongoing AKI-specific MSC trial.
NCT044 45220 Clinical Trials.gov (SBI-101)	A study of cell therapy in COVID-19 subjects with acute kidney injury who are receiving renal replacement therapy	Not specified Interventional	COVID-19 patients with AKI and sepsis requiring renal replacement therapy	SBI-101 extracorporeal MSC system (same hollow-fiber device as NCT03015623) [d] Low: 2.5 × 10^8^ cells; High: 7.5 × 10^8^ cells	Primary: Safety/tolerability (IP-related SAEs)	[Unknown] United States. No published results identified. Extension of the SBI-101 platform (NCT03015623) to COVID-19/sepsis-associated AKI context. Listed as “Unknown” status in registry.

[a] Dose per single renal artery; bilateral administration doubles the total dose. Protocol approved by the Japan Ministry of Health, Labor and Welfare (SKRM2-003). [b] NCT07101913 is an observational study, not a regenerative therapy intervention trial. Included to illustrate the nephrotoxicity risks of cell-based therapies in the ICU setting. [c] NCT01275612 was withdrawn prior to enrollment; no participants were treated. Included to document the first clinical translation attempt of MSC therapy in chemotoxic AKI. [d] SBI-101 employs an extracorporeal hollow-fiber hemofiltration device with BM-MSCs immobilized in the extracapillary space; paracrine factors are released into circulating blood without direct cell infusion, distinguishing this from conventional IV MSC administration. AKI, acute kidney injury; adMSC, adipose-derived mesenchymal stem cell; BM, bone marrow; CAR-T, chimeric antigen receptor T cell; CKD, chronic kidney disease; CRRT, continuous renal replacement therapy; eGFR, estimated glomerular filtration rate; G-CSF, granulocyte colony-stimulating factor; HB, Hope Biosciences; ICU, intensive care unit; IP, investigational product; IV, intravenous; jRCT, Japan Registry of Clinical Trials; MSC, mesenchymal stem cell; RCT, randomized controlled trial; RRT, renal replacement therapy; SAE, serious adverse event.

These observations are better understood as a reflection of clinical reality rather than a refutation of biological rationale. AKI in humans, particularly in critically ill patients, seldom behaves as a clearly defined or stable disease entity. Changes in renal function frequently occur alongside shifts in systemic physiology, evolving requirements for organ support, and competing clinical priorities. In this setting, factors unrelated to kidney-specific mechanisms often decisively determine outcomes, complicating attribution of changes in renal trajectories to a single therapeutic intervention (7, 45).

### 3.1 Post-operative AKI

Post-operative AKI, most commonly observed after cardiac surgery, was one of the first clinical contexts in which regenerative cell therapies were tested (43). The rationale for selecting this population was largely practical. Kidney injury after cardiac procedures arises within a well-defined perioperative period in highly monitored environments, with considerable AKI incidence despite surgical and perioperative improvements. Together, these features made cardiac surgery-associated AKI an accessible population for early clinical investigation.

Initial studies concentrated on MSC-based therapies, administered either prophylactically to patients considered at increased risk or therapeutically shortly after AKI was recognized. Most investigations were small, early-phase trials designed primarily to assess feasibility and safety. In most clinical studies, MSCs were administered intravenously; however, there was no consistent approach to how this was implemented. Considerable variation existed with respect to dosing schemes, timing of administration, and criteria used to select eligible patients. Treatment effects were primarily assessed using conventional clinical kidney function indicators: serum creatinine trends, AKI severity classification, RRT initiation, and short-term in-hospital outcomes (43).

When these studies are considered together, a similar overall pattern becomes apparent. MSC administration was generally safe and feasible from a practical standpoint, but a clear signal of improved renal recovery was not observed. Although some trials suggested limited benefit, signals were inconsistent across study populations and often attenuated with alternative endpoints or longer follow-up (4, 43).

Several factors likely underlie these largely neutral results. AKI following cardiac surgery does not represent a single, uniform disease process but rather a spectrum of injury mechanisms. The relative contribution of ischemia-reperfusion injury, inflammatory responses, perioperative circulatory instability, and exposure to nephrotoxic agents varies substantially between patients. This heterogeneity complicates identifying patient subgroups expected to yield predictable therapeutic effects from targeted regenerative interventions, particularly in studies with limited sample sizes (7). In addition, treatment timing may have constrained efficacy. In many trials, MSCs were administered after established renal injury, potentially too late for meaningful immune modulation (4).

Interpretation is further limited by the endpoints selected. Serum creatinine, central to routine clinical assessment, coarsely reflects renal injury and recovery, strongly influenced by fluid balance, hemodynamic status, and muscle mass. As a result, it may fail to capture the more subtle biological effects that regenerative therapies are hypothesized to exert. Furthermore, most post-operative AKI trials did not evaluate long-term renal outcomes or survival, limiting detection of clinically meaningful benefits even if present (45).

### 3.2 AKI requiring CRRT: regenerative therapy as an add-on to organ support

Different considerations apply in patients with severe AKI who require CRRT. In this group, kidney injury rarely occurs in isolation but as part of a broader clinical syndrome marked by systemic inflammation, endothelial dysfunction, and multi-organ involvement. Crucially, these patients are already supported with extracorporeal therapies, a factor that reshapes both the timing and the intended role of regenerative interventions.

Rather than focusing on direct restoration of renal function, contemporary strategies increasingly view cell-based and cell-derived therapies as adjuncts to organ support. The objective is to influence the inflammatory and immune environment that perpetuates ongoing organ dysfunction, rather than to replace damaged renal tissue itself. This conceptual shift is supported by experimental and translational evidence indicating that stromal therapies exert their principal effects through immunomodulatory and paracrine pathways (44).

The mechanistic basis for EV-mediated renal protection delivered through perfusion circuits has been established across a series of complementary experimental and translational studies. In an isolated rat DCD kidney model, hypothermic machine perfusion supplemented with MSC-derived EVs produced markedly lower global ischemic damage scores and near-complete abolition of severe tubular lesions compared to perfusion solution alone, with gene expression profiling and effluent biochemistry confirming upregulation of mitochondrial energy metabolism pathways, reduced oxidative stress markers, and preserved pyruvate levels (49). These findings were extended to a human *ex vivo* model using kidneys from extended criteria donors deemed unsuitable for transplantation, where HOPE combined with MSC-derived EVs resulted in better-preserved mitochondrial ultrastructure, higher cytochrome c oxidase IV-1 expression, reduced caspase-3 positivity, elevated HGF and VEGF tissue levels, and biochemical evidence of intact gluconeogenic capacity during normothermic reperfusion (50). Subsequent mechanistic work identified the ecto-5’-nucleotidase CD73 as a key mediator of these effects: EVs derived from CD73-silenced MSCs lost their capacity to induce tubular cell proliferation *in vitro* and failed to protect against ischemic injury *ex vivo*, while intact EV-perfused kidneys demonstrated the highest tissue ATP content and a progressive rise in effluent adenosine, consistent with CD73-driven AMP dephosphorylation replenishing cellular energy reserves through adenosine salvage pathways (51).

**FIGURE 2:**
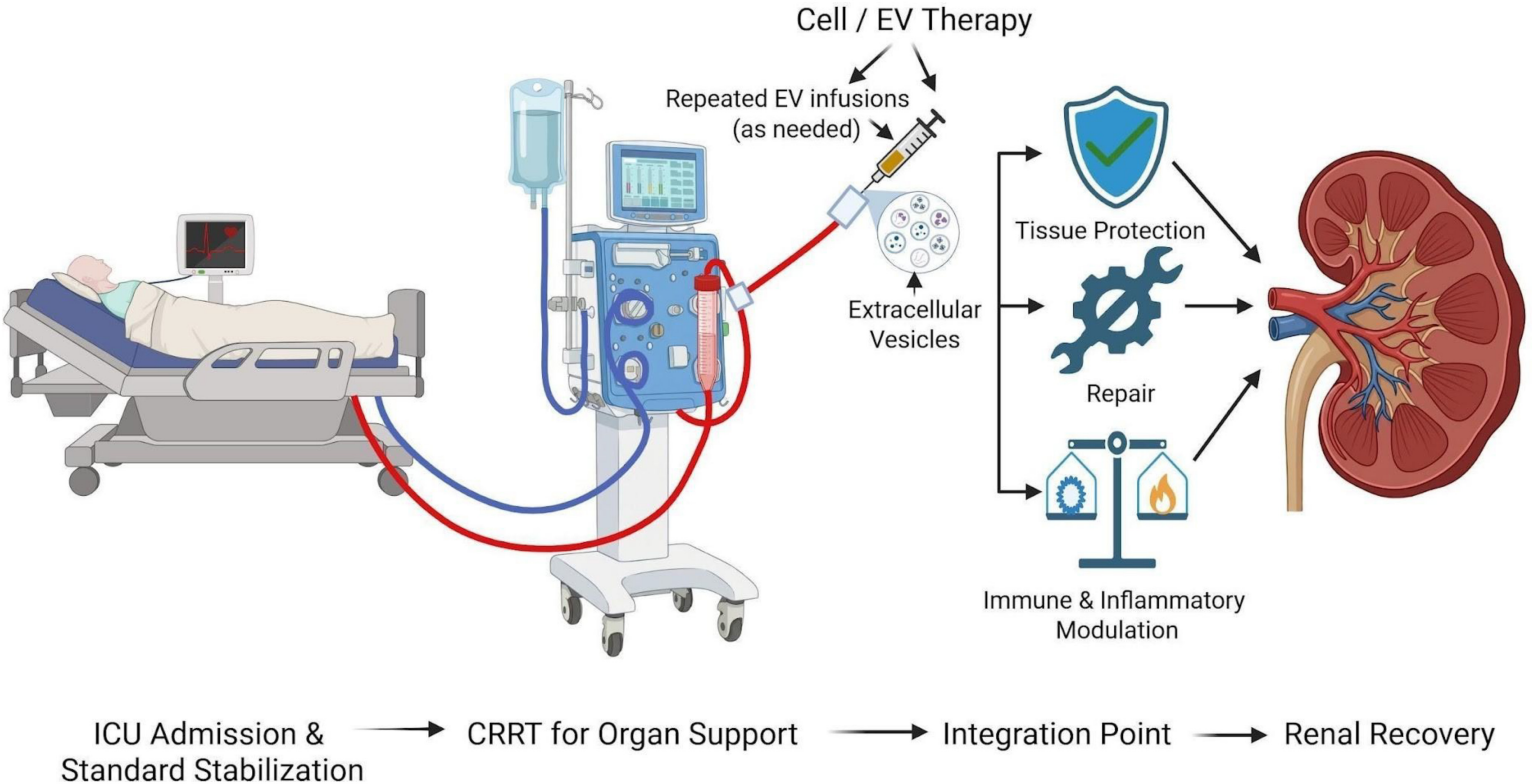
Proposed conceptual framework of clinical workflow illustrating the integration of cell/EV therapy into standard ICU management and CRRT in patients with acute kidney injury. Following ICU admission and standard stabilization, CRRT is initiated for organ support. Cell/EV therapy is administered as an adjunct via the extracorporeal circuit at a defined integration point, with the possibility of repeated EV infusions as clinically indicated. The proposed effects include modulation of immune and inflammatory responses and support of tissue protection and repair, thereby potentially supporting renal recovery.

Within this context, CRRT has emerged as a practical platform for therapeutic integration. Approaches that align delivery with extracorporeal circulation, as well as device-enabled delivery systems, have attracted growing interest. A conceptual overview of how regenerative therapy can be integrated into CRRT-supported care is illustrated in Figure 2. Early translational studies suggest that such strategies are technically achievable and safely implementable, with reports documenting changes in inflammatory biomarkers or immune cell profiles supporting their biological plausibility (17). However, as observed with other extracorporeal therapies, the expansive surface area of standard dialyzer membranes introduces a significant risk of passive therapeutic adsorption and circuit clearance, which can drastically reduce the recovery and delivery of circulating EVs (13, 15).

At the same time, the realities of intensive care impose substantial constraints. Anticoagulation protocols, hemodynamic instability, frequent circuit interruptions, and variability in CRRT practice all influence therapeutic exposure. Critically ill patients routinely receive multiple concomitant interventions that may obscure or modify regenerative therapy effects. Within such a dynamic clinical environment, the difficulty of demonstrating a consistent therapeutic signal is not unexpected. The absence of clear efficacy signals in this setting therefore reflects the complexity of human AKI rather than a failure of the underlying biological rationale (7, 45).

### 3.3 Sepsis-associated AKI / MODS cohorts

Many critical care regenerative therapy studies deliberately enrolled broad sepsis or multiple organ dysfunction syndrome (MODS) patient populations, rather than restricting inclusion to individuals with clearly defined AKI. In such cohorts, kidney dysfunction is common and clinically relevant, yet it is rarely positioned as the primary therapeutic target. Trial designs emphasize global outcomes, such as survival, organ support duration, or organ failure composites, while renal endpoints are secondary or exploratory (7).

Against this background, renal signals reported in sepsis or MODS cohorts have been variable and difficult to interpret. In some analyses, treated patients appeared to experience less severe AKI, earlier recovery of kidney function, or a reduced need for RRT. These observations are consistent with the known immunomodulatory and anti-inflammatory properties of stromal and cell-derived therapies and therefore remain biologically plausible. However, these findings often stem from *post hoc* analyses, small subgroups, or non-prespecified endpoints, limiting kidney injury-specific conclusions (4, 44).

The inherent heterogeneity of sepsis-associated AKI further complicates interpretation. The onset of kidney injury may precede, coincide with, or follow systemic infection; host immune responses vary widely; and renal trajectories are strongly influenced by hemodynamic instability and other failing organs. Without AKI-focused enrollment criteria and standardized kidney-specific endpoints, distinguishing renal recovery effects from broader systemic improvements remains challenging (7, 45). Consequently, while sepsis and MODS studies provide valuable safety and systemic biological activity data, they offer limited resolution for assessing regenerative therapy efficacy specifically for AKI.

## 4 EV Therapies: closer to “drug-like,” but clinically under-defined

EVs represent a conceptually attractive alternative to whole-cell therapies for AKI in critical care settings. Unlike live stem cells, which require careful handling, brief viability windows, and complex logistical coordination, EVs offer practical advantages that align more closely with conventional ICU workflows. These nano-sized membrane-bound particles, typically ranging from 30 to 1000 nm in diameter, can be manufactured at scale, cryopreserved for extended periods, and administered as off-the-shelf products with standardized dosing protocols (52). Furthermore, EVs carry reduced theoretical risks compared to cellular therapies, including the absence of tumorigenic potential, lower immunogenicity, and minimal concerns regarding pulmonary entrapment or unwanted engraftment (53). These characteristics position EVs as potentially more “drug-like” therapeutics that could be integrated into existing critical care protocols without the infrastructure demands inherent to cell-based interventions. The biological rationale for EV therapy in AKI builds upon extensive preclinical evidence demonstrating renal protective and regenerative effects. MSC-derived EVs have been shown to reduce tubular injury, modulate inflammation, promote epithelial proliferation, and restore microvascular perfusion in animal models of ischemic, post-surgery, and septic AKI (54, 55). These effects appear mediated through horizontal transfer of microRNAs, mRNAs, proteins, and lipids that reprogram recipient cells toward pro-repair phenotypes (4, 54). Unlike whole cells, EVs can potentially access injured tissue more readily due to their smaller size and may avoid some immune recognition mechanisms that limit allogeneic cell therapies. Whether these theoretical advantages translate into clinically meaningful benefits depends on resolving key translational challenges, including scalability of GMP (good manufacturing practice) manufacturing, product standardization, safety characterization, and regulatory definition.

Successfully translating EV therapies from preclinical promise to ICU workflows requires a systematic evaluation across four primary pillars: scalability, product standardization, safety profiles, and regulatory feasibility. In terms of scalability and logistical integration, whole-cell therapies face inherent logistical constraints in ICU settings, requiring real-time manufacturing coordination, narrow viability windows, and specialized handling that limits their scalability across centers. EVs circumvent many of these barriers. From an operational perspective, EVs offer distinct advantages for ICU implementation. Dosing can be weight-based and rapidly prepared, similar to conventional pharmacotherapies. The absence of living organisms reduces concerns about product viability during transport and bedside delays. Moreover, EVs can be administered via standard intravenous access without specialized infusion protocols, lowering the barrier to adoption across multiple ICU settings. These practical features make EVs inherently more scalable than cell therapies, potentially enabling broader access if efficacy is demonstrated.

### 4.1 Extracellular vesicles storage and dose for repetitive transplantation

Manufacturing challenges compound these scientific uncertainties. Good manufacturing practice-compliant production of EVs at clinical scale requires optimization of multiple process steps. Bioreactor cultivation of parent cells must be standardized for cell density, culture duration, media composition, and environmental conditions, as each parameter influences EV yield and cargo composition (56). Storage conditions critically influence EV stability and bioactivity, with emerging data suggesting that freeze-thaw cycles, storage temperature, and formulation buffers significantly affect membrane integrity, particle aggregation, and cargo retention (57). Storage at −80 °C maintains EV stability for months, eliminating the real-time coordination required for fresh cell products (57). While freezing at - 80°C appears to preserve many EV functions, but some studies report loss of specific activities after long-term storage (57). Lyophilization has been explored as an alternative but requires careful optimization of cryoprotectants and reconstitution protocols.

Regarding product standardization, cell therapies, particularly MSC-based products, have made incremental regulatory progress in defining identity and potency criteria. For EVs, the standardization landscape remains considerably less mature. Despite these conceptual advantages, the translation of EV therapies from preclinical promise to bedside reality remains stymied by fundamental gaps in product characterization and standardization. The ISEV (International Society for Extracellular Vesicles) has established Minimal Information for Studies of Extracellular Vesicles (MISEV) guidelines, most recently updated in 2023, yet substantial heterogeneity persists in EV isolation methods, characterization techniques, and quality control measures across research groups (58). This variability directly impacts product definition: what constitutes a therapeutic “dose” of EVs remains poorly standardized, with investigators reporting quantities in total protein mass, particle number by nanoparticle tracking analysis, RNA content, or parent cell equivalents without universal consensus (59). The lack of validated potency assays specific to renal repair represents a critical bottleneck. While *in vitro* assays may measure generic properties such as anti-inflammatory cytokine profiles, uptake by target cells, or proliferation-inducing capacity, these surrogate markers do not necessarily predict *in vivo* efficacy for AKI treatment (59). Nevertheless, these gaps reflect the field’s early stage rather than fundamental insurmountability, and EV standardization challenges are arguably more tractable than the biological variability inherent to live cell products.

### 4.2 Safety, regulatory, and trial design considerations

Evaluating safety profiles reveals that, compared to whole-cell therapies, EVs eliminate risks of tumorigenic potential, pulmonary embolism, and ectopic engraftment. However, this does not render them categorically safer; rather, they carry a distinct and less well-characterized risk profile. Safety surveillance constitutes another essential but under-addressed priority. Although EVs lack the proliferative capacity of intact cells, they are not inherently inert. EVs can transfer functional molecules, including microRNAs, proteins, metabolites, and lipids that may exert unintended biological effects (52). Preclinical studies have documented both beneficial and potentially adverse immunomodulatory effects depending on EV source, dose, recipient inflammatory state, and disease context (54, 60). Nonetheless, these cargo-mediated risks remain more amenable to characterization and mitigation than the engraftment and embolism risks associated with whole-cell administration in critically ill patients.

In terms of regulatory feasibility, cell therapies have established regulatory pathways, the RMAT (regenerative medicine advanced therapy) designation of FDA (Food and Drug Administration), and the ATMP (Advanced Therapy Medicinal Products) framework of EMA (European Medicines Agency), that provide structured development routes. EVs currently lack equivalent clarity, occupying an ambiguous classification space that creates additional uncertainty for trial design and market authorization. These manufacturing complexities have practical regulatory implications, as agencies including the FDA and EMA increasingly scrutinize product specifications, release criteria, stability data, and comparability assessments before permitting first-in-human studies (57). For AKI-focused EV trials to yield interpretable and clinically meaningful results, several design elements are paramount. First, enrollment criteria must specifically target AKI as the primary condition of interest rather than including renal dysfunction as an incidental feature of general critical illness. The Kidney Disease: Improving Global Outcomes (KDIGO) staging system provides a standardized framework for AKI diagnosis and severity classification based on serum creatinine changes and urine output that should be uniformly applied across trials (61).

Beyond KDIGO staging, enrichment strategies using novel biomarkers such as urinary NGAL (Neutrophil Gelatinase-Associated Lipocalin), TIMP-2 and IGFBP7 (Tissue inhibitor of metalloproteinase-2 and insulin-like growth factor binding protein 7), or kidney injury molecule-1 may identify patients at the highest risk for progression who are most likely to benefit from intervention (62). Second, endpoint selection must move beyond generic mortality outcomes toward renal-specific measures that capture meaningful recovery. While all-cause mortality remains important, it lacks sensitivity for detecting renal-specific benefits and may be influenced by numerous non-renal factors in critically ill populations. Dialysis-free days, a composite metric reflecting both survival and freedom from renal replacement therapy, offers a clinically relevant endpoint increasingly adopted in critical care nephrology trials (45). This endpoint accounts for competing risks of death and dialysis while incorporating the duration of independence from renal support. Major adverse kidney events at 30 or 90 days, encompassing death, new dialysis dependence, and sustained reduction in estimated glomerular filtration rate of ≥25%, provide another validated composite outcome that captures both survival and kidney function (45). Incorporation of both short-term recovery metrics and longer-term functional outcomes would comprehensively assess therapeutic impact across relevant time horizons. Short-term measures should include urine output trends, serum creatinine trajectory, need for and duration of renal replacement therapy, and novel biomarker kinetics reflecting tubular injury and repair. Longer-term follow-up at 3, 6, and 12 months should assess eGFR recovery, incident chronic kidney disease, quality of life measures, and healthcare resource utilization including hospital readmissions and dialysis dependence. Such multi-dimensional endpoint frameworks provide richer data for regulatory decision-making and clinical translation. Despite this ambiguity, the absence of live organism classification may ultimately simplify certain aspects of EV regulation compared to somatic cell therapy requirements.

## 5 Conclusion and future perspectives: practical roadmap

The path toward clinically meaningful cell and EV therapies for AKI in critical care requires confronting both biological complexity and pragmatic implementation challenges. Near-term success is most likely in carefully selected use cases where patient homogeneity is greatest and intervention timing can be standardized. Patients receiving CRRT for severe AKI represent one such population: enrollment criteria can be tightly defined, intervention timing relative to circuit initiation can be protocolized, and renal-specific endpoints are directly relevant (63). Several fundamental barriers must be systematically addressed. AKI heterogeneity spanning diverse etiologies, severity stages, and recovery trajectories demands enrichment strategies targeting specific phenotypes (63). Five action-oriented priorities should guide next-generation studies: First, establish multicenter networks achieving adequate power while maintaining phenotype-specific enrollment. Second, develop validated AKI-specific potency assays predicting renal repair capacity. Third, conduct dedicated pharmacokinetics/pharmacodynamics studies in critically ill patients, including circuit interaction modeling. Fourth, implement adaptive platform trial designs enabling efficient evaluation of multiple products and subgroups. Fifth, embed mechanistic substudies, such as biomarker kinetics, imaging, and transcriptomics, to elucidate response predictors and optimize future iterations.
